# Loss and recovery of neurofilament in the cat lateral geniculate nucleus following monocular retinal inactivation

**DOI:** 10.3389/fnins.2026.1802413

**Published:** 2026-03-31

**Authors:** Andrea De Paola, Robert W. Jones, Jonathon M. Henneberry, Kevin R. Duffy

**Affiliations:** Department of Psychology and Neuroscience, Dalhousie University, Halifax, NS, Canada

**Keywords:** monocular inactivation, retinal inactivation, dLGN, visual cortex, neurofilament, monocular deprivation, neural plasticity, critical period

## Abstract

**Background:**

Postnatal development of the mammalian primary visual pathway occurs early in life and is guided by visually-driven afferent activity. Even a short duration of disrupted binocular vision can derail development of neural connections and produce a lasting monocular visual impairment, amblyopia. Temporary inactivation of the dominant retina with tetrodotoxin has emerged as a potential treatment for amblyopia that has exhibited superior potency compared to mainstay therapy in animal models. Notwithstanding its promise as a treatment for amblyopia, little is known about the impact of retinal inactivation on neurons within the primary visual pathway.

**Methods:**

We investigated the impact that monocular inactivation (MI) imposed at different postnatal ages has on neurofilament immunolabeling within eye-specific layers of the cat dorsal lateral geniculate nucleus (dLGN). Neurofilament is a constituent of the stable neuronal cytoskeleton and a sensitive marker for different kinds of visual deprivation that produce amblyopia. A comparison of the potential for neurofilament recovery was also examined after either MI or monocular deprivation (MD).

**Results:**

Data revealed a profound efficacy for MI to elicit reduction of neurofilament labeling in dLGN layers serving the inactivated eye. The effect of MI was greatest when administered early in development near the critical period peak, then declined considerably with age, though an effect was still observed at the oldest age examined. Despite the significant loss of neurofilament produced by MI, near complete recovery was measured when binocular vision was provided after the period of inactivation but this was not observed after MD.

**Conclusion:**

These results demonstrate the remarkable efficacy of MI to elicit significant modification of neurofilament within the dLGN throughout early postnatal development, and they also reveal a surprising capacity for recovery following the period of inactivation.

## 1 Introduction

Development of neural circuitry within the mammalian primary visual pathway is guided by stimulus-driven afferent impulses (Wiesel and Hubel, 1963a; Crair, 1999). Manipulation of retinal output activity during early postnatal development can obstruct the maturation of neurons and the arrangement of their connections (Wiesel and Hubel, 1963b; Mioche and Singer, 1989). Monocular eyelid closure (i.e., MD) is a common experiential manipulation of vision that produces an imbalance in retinal activity between the eyes. The interocular mismatch in afferent activity caused by MD triggers a litany of neurodevelopmental modifications that manifest throughout the primary visual pathway (Duffy et al., 2023a), and which are enabled by a constellation of plasticity-related molecules that govern the capacity for neural modification (Murphy et al., 2005; Murphy and Monteiro, 2024). Even a short duration of MD imposed early in postnatal development can precipitate weakening and even elimination of deprived-eye synapses that shifts cortical ocular dominance in favor of the non-deprived eye (Wiesel and Hubel, 1963a; Tieman, 1984; Bear and Colman, 1990). Within the cat dLGN, MD early in postnatal life reduces the size of neurons in layers postsynaptic to the deprived eye compared to non-deprived counterparts (Wiesel and Hubel, 1963b; Duffy et al., 2015). An acutely sensitive neuroanatomical marker for revealing the impact of visual deprivation is loss of neurofilament protein within deprived-eye layers of the dLGN (Bickford et al., 1998; Duffy and Slusar, 2009; Duffy et al., 2014; Shkorbatova et al., 2022) and within deprived-eye ocular dominance columns of the primary visual cortex (Fenstemaker et al., 2001; Duffy and Livingstone, 2005; Duffy et al., 2007; Zehra et al., 2026).

Neurofilament is a neuron-specific developmentally regulated cytoskeletal protein that is enriched within projection neurons (Duffy et al., 2012; Song et al., 2015). It is a key constituent of axonal volume (Cleveland et al., 1991) and is critical for the growth and maintenance of normal axon caliber and neuron conduction velocity (Nixon et al., 1994; Perrot et al., 2007; Nowier et al., 2023). The assembly of neurofilament into an intracellular scaffold confers long-term structural stability to the network of neural connections cultivated by visual experience during the critical period (Smith, 1973; Song et al., 2015; van Asperen et al., 2024). The physiological and anatomical modifications elicited by MD that include loss of neurofilament are associated with a vision impairment, amblyopia, characterized by reduced spatial acuity in the deprived eye as well as loss of binocularity (Giffin and Mitchell, 1978; Vorobyov et al., 2007; Birch and Duffy, 2024). The perturbations catalyzed by MD demonstrate the dynamic nature of neurons within the developing visual system, and underscore the important role that visually-driven impulse activity can play in the development of neurons and the organization of neural connections within and beyond the primary visual pathway (Dooley and van der Heijden, 2025).

While modifications induced by MD demonstrate the importance of visually-driven activity, complete inactivation of retinal afferent impulse conduction with a voltage-gated sodium channel blocker, tetrodotoxin (TTX), has revealed the impact that spontaneous non-visually-driven activity can have on visual system development (Duffy et al., 2023b). In cats, blockade of retinal output activity in one eye with TTX application elicits a host of neural modifications that include a decrease in oxidative metabolism (Wong-Riley and Riley, 1983), and a significant reduction in the size of dLGN neurons within inactivated-eye layers (Kupperman and Kasamatsu, 1983; Duffy et al., 2023a). These changes appear to arise at least in part from the absence of orthodromic activity rather than from binocular competition alone (Duffy et al., 2023b). Although changes elicited by MI in cats are pronounced, and significantly larger than what is produced by a comparable period of MD, the alterations are reversed when the influence of TTX wanes and binocular vision is restored (Kupperman and Kasamatsu, 1983; Wong-Riley and Riley, 1983). Effects of TTX-induced MI have also been documented in monkeys. Following protracted MI, affected layers of the monkey dLGN and cortical ocular dominance columns serving the inactivated eye exhibit reduced cytochrome oxidase staining and *c-fos* expression (Wong-Riley and Carroll, 1984; Hendry and Miller, 1996; Takahata and Kaas, 2017), as well as loss of immunolabeling for gamma-aminobutyric acid (GABA) neurotransmitter and its synthesizing enzyme, glutamic acid decarboxylase (Hendry and Jones, 1988). After cessation of TTX and restoration of binocular vision, changes elicited by MI in monkeys can also recover to normal levels (Hendry and Jones, 1988). The transient nature of MI effects suggest that recovery is catalyzed by the restoration of afferent activity, which raises a possible dichotomy between MI and MD effects in relation to their longevity and potential for recovery.

In the current study, we examined the eye-specific layers of cat dLGN to determine if the level of neurofilament protein was selectively altered by eliminating spontaneous retinal activity in one eye with intravitreal administration of TTX. The effect of MI on neurofilament labeling was assessed at different ages throughout postnatal development. We also compared the capacity for neurofilament recovery when MI and MD were followed by binocular vision. We observed that MI produced a reduction of neurofilament labeling within layers of the dLGN postsynaptic to the inactivated eye, and the effect of MI adhered to a critical period in which younger animals expressed the largest effects. Following MI, affected layers recovered near normal levels of neurofilament immunolabeling when inactivation wore off and binocular vision was restored, whereas less recovery was observed following a comparable loss of neurofilament produced by MD.

## 2 Methods

### 2.1 Animals

**Figure 1:**
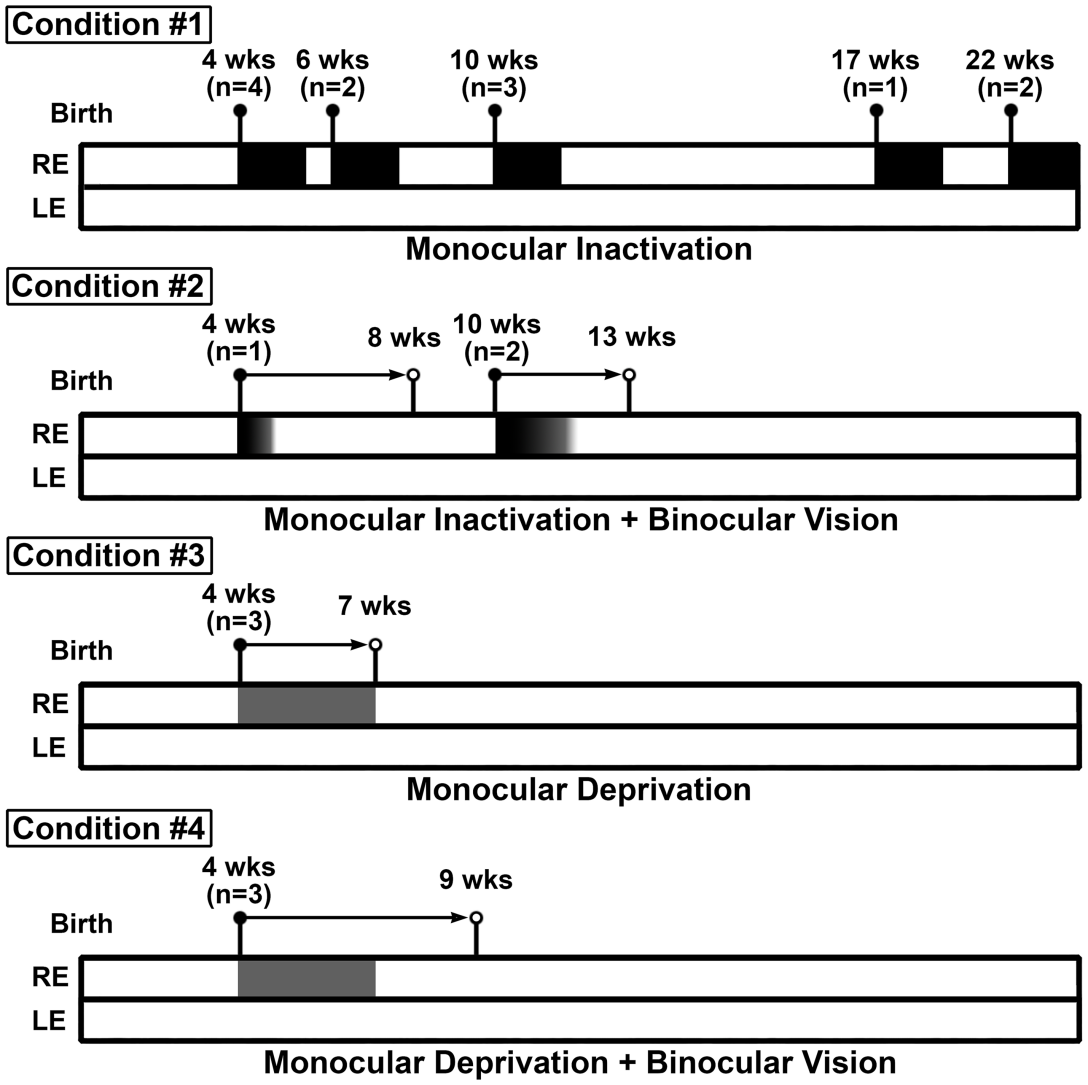
Schematic depicting the timing of visual manipulations and rearing conditions of animals in this study. For condition #1, all animals received 10 days of MI initiated at the ages indicated and did not receive subsequent binocular vision. For condition #2, one animal received approximately 5 days of MI at 4 weeks of age followed by binocular vision until 8 weeks of age, and a separate group of animals received approximately 13 days of MI followed by a week of binocular vision. Animals in condition #3 were monocularly deprived for 3 weeks starting at 4 weeks of age and received no binocular vision. For condition #4, animals were monocularly deprived for 3 weeks starting at 4 weeks of age, then had 2 weeks of binocular vision.

Studies were conducted on 21 cats that were born and raised in a closed colony at Dalhousie University. All tissues used for this investigation were obtained from previously studied animals that are inventoried within our cat brain tissue bank (Aronitz et al., 2021; Henneberry et al., 2023). Rearing protocols and experimental procedures were conducted in compliance with guidelines established by the University Committee on Laboratory Animals at Dalhousie, as well as those detailed by the Canadian Council on Animal Care. This study examined the effect of 10 days of MI imposed at various ages across postnatal development that ranged from 4 to 22 weeks (*n* = 12). To assess recovery from the effect of MI, a group of animals received a period of MI at 4 (*n* = 1) or 10 (*n* = 2) weeks of age followed by binocular vision. To compare the capacity for recovery between MI and MD, a group of animals received 3 weeks of MD by eyelid closure (*n* = 3), and a separate group received the same MD followed by a period of binocular vision (*n* = 3). The rearing history of each animal is presented in Table 1, and a schematic of the study design is presented in Figure 1. Although the number of animals used in this study is typical for the species, sample sizes are usually small for cat studies because of inherent constraints such as smaller litter sizes, longer gestational times, and a more protracted critical period compared to rodents.

**Table 1:** Animal rearing conditions.

	Rearing manipulations			
	Normal	Inactivation	MD	BV
Condition #1 Monocular inactivation				
*n* = 4	P0–P30	P30–P40	–	–
*n* = 2	P0–P42	P42–P52	–	–
*n* = 3	P0–P70	P70–P80	–	–
*n* = 1	P0–P119	P119–P129	–	
*n* = 2	P0–P154	P154–P164	–	–
Condition #2 Monocular inactivation + BV				
*n* = 1	P0–P30	P30–P35	–	**P35–P56
*n* = 2	P0–P70	P70–P83	–	**P83–P90
Condition #3 Monocular deprivation				
*n* = 3	P0–P30	–	P30–P51	–
Condition #4 Monocular deprivation + BV				
*n* = 3	P0–P30	–	P30–P51	P51–P65

Age in postnatal days is indicated by P. BV refers to binocular vision. **The duration of binocular vision is an estimate based on the finding that vision fully recovers about 5 days after TTX injection (Kwaw et al., 2025). The animal in Condition #2 that received MI at 4 weeks of age then binocular vision to 8 weeks of age is estimated to have had about 5 days of inactivation because it was administered only one intravitreal TTX injection.

### 2.2 Monocular inactivation

Animals that received MI were first anesthetized with 3–4% isoflurane and local anesthetic drops (1% proparacaine hydrochloride) were administered to the right eye before delivering an intravitreal microinjection of 3 mM TTX (ab120055; abcam, United States) solubilized in citrate buffer. For each animal, the dosage of TTX was calculated according to eye size linked with age (Thorn et al., 1976). We injected 0.5 μL of TTX per mm of vitreous chamber length. This approximate dosage is known to block action potentials of affected cells without obstructing critical cellular functions (Ochs and Hollingsworth, 1971). Injections were delivered through a very small puncture that was made in the sclera located at the pars plana. Using a surgical microscope, the full volume of TTX solution was dispensed into the vitreous chamber using a sterilized Hamilton syringe (Hamilton Company, United States) fitted with a 30-gage needle (point style 4). The needle was positioned through the original puncture and about 5–10 mm into the chamber angled away from the lens. The complete volume of TTX was injected slowly, and, once complete, the needle was kept in place for about a minute before it was retracted. Following intraocular injection, topical antibiotic (1% chloromycetin) and a local anesthetic (1% proparacaine hydrochloride) were applied to the eye to prevent post-injection complications. The anti-inflammatory, Metacam (meloxicam; 0.05 mg/kg), was also provided for post-procedure analgesia. To ensure 10 days of complete inactivation, animals received five total injections, each separated by 48 h. Following the first injection, the original puncture site was used for subsequent injections to avoid having to make another hole in the sclera. Retinal inactivation was evident by the emergence of an anisocoria due to mydriasis in the injected eye. This resolved when the effect of TTX wore off. In a collection of animals, retinal inactivation was verified by the absence of a pupillary light reflex as well as the lack of visuomotor behaviors such as visual placing, visual startle, and the ability to track a moving laser spot while the seeing eye was occluded.

### 2.3 Monocular deprivation

Animals were monocularly deprived while under general gaseous anesthesia (3–4% isoflurane in oxygen) and it was implemented by closure of the upper and lower palpebral conjunctivae with sterile 5–0 vicryl, followed by closure of the eyelids with 5–0 silk suture. Once the procedure was completed, animals were administered oral Metacam (0.05 mg/kg) for post-procedure analgesia, local anesthesia was produced with drops of Alcaine sterile ophthalmic solution (1% proparacaine hydrochloride; CDMV, Canada), and infection was mitigated with application of a broad-spectrum topical antibiotic (1% chloromycetin; CDMV). Eye closure was monitored each day to ensure the lids were in good health and fully closed. The group of animals whose MD was followed by a period of binocular vision had their deprived eye opened under gaseous anesthesia (3–4% isoflurane in oxygen) by removing the sutures. To minimize the incidence of post-procedure infection, the prophylaxis protocol used during the initial eyelid closure was again followed.

### 2.4 Tissue preparation

In preparation for histological processing, animals were euthanized with a lethal dose of sodium pentobarbital (Pentobarbital Sodium; 150 mg/kg) and then were exsanguinated by transcardial perfusion with approximately 150 mL of phosphate buffered saline (PBS) followed by an equivalent volume of PBS containing 4% dissolved paraformaldehyde. Following perfusion, brain tissue was extracted and the thalamus was resected to prepare the dLGN for sectioning and histological processing. Tissue containing the dLGN was first cryoprotected by immersion in a PBS solution containing 30% sucrose, then was cut coronally into 50 μm sections. Tissue slices were stored at −20 °C while immersed in an antigen preservative solution (Burke et al., 2009) until used for this study. Tissue sections processed for this study were all subjected to the same extraction process, preparation procedures, storage settings, and all sections were subjected to the same immunolabeling protocol.

### 2.5 Histology

For each animal, six sections containing the left and right dLGN were selected for neurofilament immunolabeling. Tissue sections were first washed in PBS, then were placed free-floating in a PBS solution containing a mouse monoclonal antibody (1:1000) targeted against the non-phosphorylated heavy chain neurofilament protein (SMI-32, RRID: AB_509998; Biolegend, San Diego, CA) and left for 12 h. After being washed with PBS, sections were immersed for 1 h in a PBS solution containing biotinylated goat anti-mouse antibody (1:500) (115-065-003; Jackson ImmunoResearch, West Grove, PA). Sections were rinsed again with PBS and then immersed in a PBS solution containing avidin and peroxidase-conjugated biotin (PK6100; Vector Laboratories, Burlingame, CA) and left for 1 h. Immunolabeling was made visible by exposure of sections to a PBS solution containing hydrogen peroxide (1:1000) and the chromogen, 3,3′-diaminobenzidine (0.5 mg/ml). After washes with PBS, sections were mounted onto glass slides and permitted to air dry, then were dehydrated using a graded series of ethanols before sections were cleared with Histo-Clear (National Diagnostics, Atlanta, GA). Slides were coverslipped with Permount mounting medium.

### 2.6 Quantification and analysis of anatomy

Sections containing the dLGN that were labeled for neurofilament were imaged with an Olympus VS200 slide scanner at high magnification using a 20x objective. Measurements for this study were performed blind to each animal’s rearing condition. Using QuPath bioimaging analysis software (Bankhead et al., 2017), the density of neurofilament positive cells was measured from layers of the left and right dLGN that receive input from either the contralateral eye (A layers) or the ipsilateral eye (A1 layers). Neurons with distinct labeling within the cytoplasm and proximal dendrites, but not within the nucleus, were counted within delineated regions of A and A1 layers. The density of neurofilament-positive cells was calculated for each layer separately by dividing cell counts by the area of each layer that was sampled. Density measurements were averaged for A and A1 layers serving each eye. Cell caps were excluded from our density counts by only selecting cells with distinct cytoplasmic labeling and weak or absent labeling within the nucleus. Assessments and measurements of the dLGN were made from sections that spanned coronal plane 6–7 (Sanderson, 1971), which is positioned about midway along the anterior–posterior axis of the nucleus.

The effect of MI and MD within each study group was assessed using a non-parametric Wilcoxon test (paired, two-tailed) that compared density measurements between eye-specific layers. For statistics, measurements from right-eye and left-eye dLGN layers from each animal were treated as independent observations. Comparison of the magnitude of the effect of deprivation between groups was achieved with an ocular dominance metric that was calculated for each animal. This calculation revealed the percentage difference between eye-specific dLGN layers for each animal.

## 3 Results

**Figure 2:**
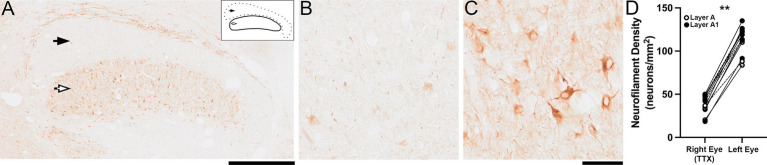
Profound loss of neurofilament in the dLGN after 10 days of MI imposed at the critical period peak. Following MI at 4 weeks of age there was a distinct loss of neurofilament immunoreactivity within layers of the dLGN postsynaptic to the inactivated eye [black arrow in **(A)**] which contrasted the much higher level of labeling observed in normal-eye layers [white arrow in **(A)**]. This effect was particularly evident with higher magnification views that revealed few immunoreactive cell bodies and processes within the inactivated-eye layer **(B)** when compared to those of the normal eye **(C)**. Results from the quantification of neurofilament-positive cell density **(D)** supported these observations by revealing a significant reduction within layers serving the inactivated eye relative to those serving the normal eye at age 4 weeks (data connected with a solid line) plotted alongside data from animals that received the same MI at 6 weeks (data connected with a dashed line). For each animal, cell density was calculated separately for the A (white circles) and A1 (black circles) layers of the dLGN. The lines in **(D)** connect layer-specific measurements for the right and left eye in the same animal. Scale bars = 1 mm **(A)**; and 100 μm **(B,C)**. Inset in **(A)** delineates layer boundaries of the left dLGN. Asterisks indicate significance less than 0.01.

A clear effect of 10 days of MI was observed in the dLGN when initiated at 4 weeks of age, coinciding with the peak of critical period plasticity (Olson and Freeman, 1980). Layers of the dLGN postsynaptic to the inactivated eye were almost completely void of neurofilament immunoreactivity (Figure 2A) apart from a smattering of immunopositive cell bodies that were evenly distributed across the layer. At high magnification, the effect of MI was also obvious with overall weak immunolabeling throughout inactivated-eye layers (Figure 2B) while layers serving the normal fellow eye had many immunoreactive cell bodies and processes (Figure 2C). Quantification of neurofilament-positive cell density (Figure 2D) following 10 days of MI at 4 weeks (symbols connected by solid lines) and also at 6 weeks of age (symbols connected by dashed lines) revealed a combined 66% reduction within inactivated-eye layers of the dLGN (mean = 38 cells/mm^2^; SD = 11 cells/mm^2^) compared with normal layers (mean = 112 cells/mm^2^; SD = 16 cells/mm^2^). This reduction in neurofilament-positive cell density after MI was statistically significant (Wilcoxon test; *p* < 0.01).

**Figure 3:**
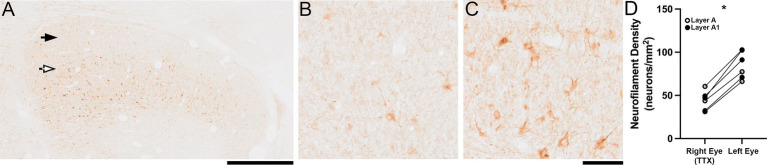
The impact of 10 days of MI on neurofilament labeling when initiated at 10 weeks of age. A conspicuous reduction in neurofilament immunolabeling was evident in dLGN layers **(A)** serving the inactivated eye (black arrow) relative to those under control of the normal eye (white arrow). Upon microscopic inspection, fewer immunoreactive cell bodies and processes were found within inactivated layers **(B)** whereas these characteristics were plentiful within normal layers **(C)**. Quantification of neurofilament-positive cell density indicated that there was a significant reduction within inactivated-eye dLGN layers compared to normal-eye layers **(D)**. Scale bars = 1 mm **(A)** and 100 μm **(B,C)**. Symbols in **(D)** represent measurements from the A (white circles) and A1 (black circles) layers, and lines connect layer-specific measurements between the eyes from the same animal. Images shown are from the left dLGN. Asterisk indicates significance less than 0.05.

The impact of 10 days of MI imposed at 10 weeks of age was less than that observed at younger ages but was still clearly visible at low magnification (Figure 3A). Notably, this reduction of neurofilament immunolabeling within inactivated-eye dLGN layers occurred weeks beyond the critical period peak when plasticity capacity has lowered. Layers of the dLGN postsynaptic to the inactivated eye showed a loss of immunoreactive cell bodies and processes (Figure 3B) that was especially clear when compared with normal-eye layers that showed high reactivity (Figure 3C). Measurement of neurofilament-positive cell density mirrored our qualitative observations by showing a 48% reduction within inactivated-eye dLGN layers (mean = 44 cells/mm^2^; SD = 11 cells/mm^2^) when compared with those serving the normal eye (mean = 85 cells/mm^2^; SD = 16 cells/mm^2^; Figure 3D). This loss of immunolabeling with MI started at 10 weeks of age was statistically significant (Wilcoxon test; *p* < 0.05).

**Figure 4:**
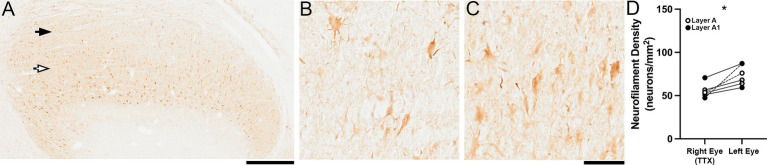
Moderate reduction of neurofilament labeling when MI was initiated in older animals aged beyond the classical critical period. A small loss of neurofilament immunoreactivity was observed following 10 days of MI started at 22 weeks of age. Layers of the dLGN serving the inactivated eye (black arrow) and normal eye (white arrow) both had strong neurofilament reactivity within cell bodies and processes **(A)**. At high magnification it was clear that there were fewer immunoreactive features within inactivated-eye layers **(B)** relative to those of the normal eye **(C)**. Measurements of neurofilament-positive cell density revealed that the reduction within inactivated-eye layers was significant. Scale bars = 1 mm **(A)** and 100 μm **(B,C)**. Symbols in **(D)** represent measurements from the A (white circles) and A1 (black circles) layers, and lines connect layer-specific measurements between the eyes from the same animal. Images shown are from the left dLGN. Asterisk indicates significance less than 0.05.

The effect of MI was clearly less as the age at the time of inactivation increased even further past the critical period peak. Inactivation for 10 days starting at 22 weeks of age elicited a reduction in neurofilament immunolabeling that was much less obvious than in animals that received MI earlier in development (Figure 4A). Still, at 22 weeks of age the loss of neurofilament was apparent within inactivated dLGN layers (Figure 4B) relative to layers serving the normal eye (Figure 4C). Unlike at younger ages, inactivated-eye layers exhibited plenty of strongly immunoreactive cell bodies and labeled processes throughout the layer. Quantification of neurofilament-positive cell density (Figure 4D) within the dLGN of animals that received 10 days of MI at 17 weeks (symbols connected by dashed lines) and 22 weeks of age (symbols connected by solid lines) revealed a combined 26% reduction in layers serving the inactivated eye (mean = 55 cells/mm^2^; SD = 8 cells/mm^2^) compared to those post-synaptic to the normal eye (mean = 74 cells/mm^2^; SD = 12 cells/mm^2^). This loss of neurofilament-positive cell density was statistically significant (Wilcoxon test; *p* < 0.05).

**Figure 5:**
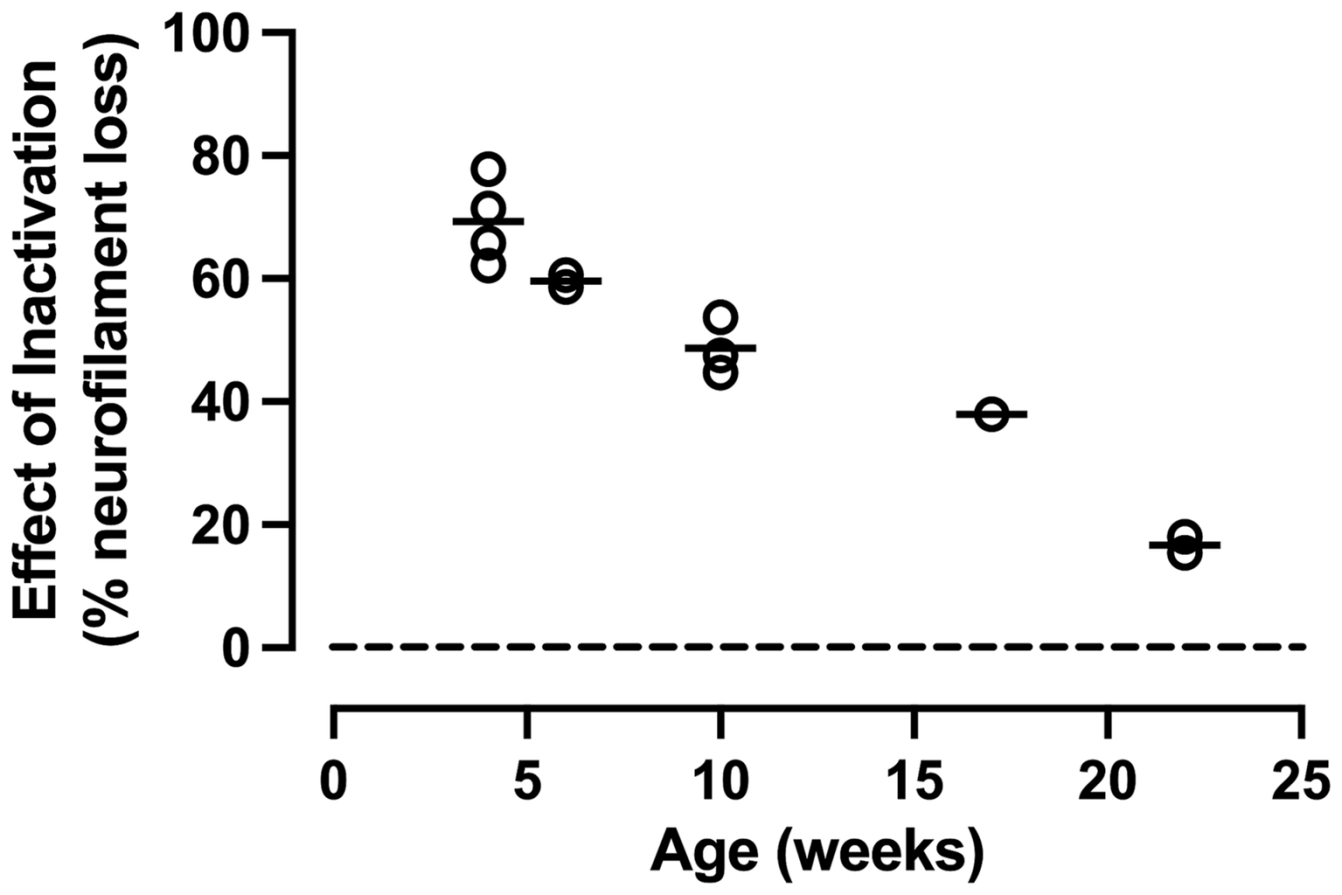
Impact of MI on neurofilament labeling in the dLGN diminishes with increasing age of onset. Whereas a profound loss of neurofilament-positive cell density was observed when MI was initiated at 4 weeks of age, the loss progressively lessened with increasing age up to 22 weeks, the oldest age examined. Notwithstanding the reduction of MI efficacy with age, inactivation continued to produce a clear reduction of neurofilament even when applied at 22 weeks old.

For each animal, an ocular dominance metric was used to calculate the percentage loss of neurofilament-positive cell density within dLGN layers serving the inactivated eye compared to the normal eye, and this was plotted as a function of the age at which MI was imposed (Figure 5). These data revealed a progressive (roughly linear) decline in the impact of MI with advancing age. The effect of 10 days of MI was reduced from 69% at 4 weeks of age to 17% at 22 weeks of age, representing a ~ 3% loss of efficacy per week. Despite this reduced sensitivity to MI, an effect was still observed at the oldest ages examined (17 and 22 weeks), which is a time when ocular dominance plasticity elicited by MD is negligible (Olson and Freeman, 1980).

The loss of neurofilament within dLGN layers serving the inactivated eye is reminiscent of other neural modifications reported to occur following MI in cats. These include a marked atrophy of neuron soma size (Kupperman and Kasamatsu, 1983; Duffy et al., 2023b) and a loss of cytochrome oxidase staining within inactivated-eye layers (Wong-Riley and Riley, 1983). Notwithstanding the large magnitude of these MI-induced changes, they appear to be non-degenerative and reversible because both deficits recover once the effects of TTX wear off. We therefore next examined the extent to which MI-induced neurofilament loss could recover with subsequent provision of binocular vision. We also assessed whether the extent of neurofilament recovery after MI was different than that observed after a period of MD that elicited a comparable loss of neurofilament.

**Figure 6:**
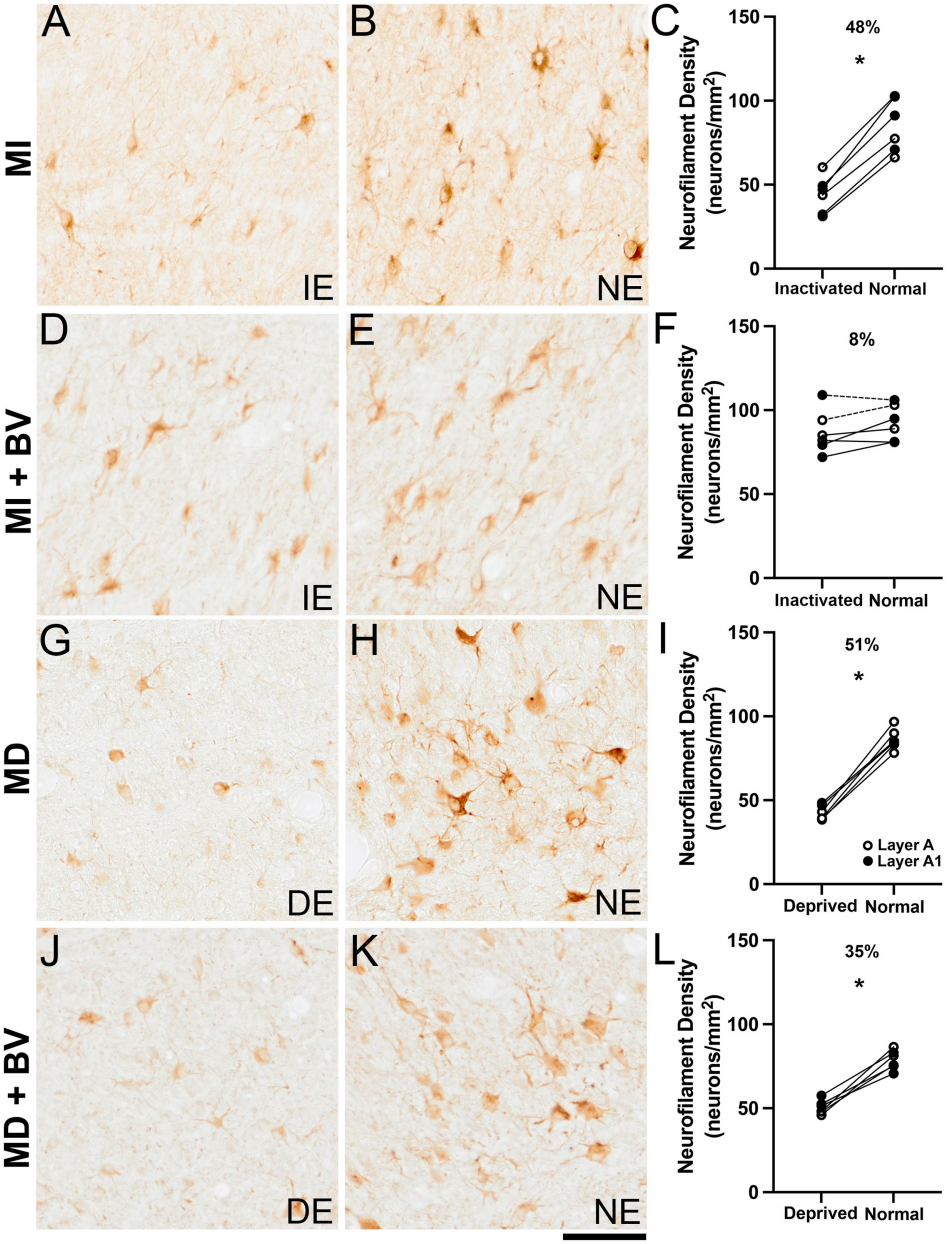
Near complete recovery of neurofilament labeling when binocular vision followed MI but not MD. Whereas 10 days of MI initiated at 10 weeks of age elicited a significant loss of neurofilament within inactivated-eye dLGN layers **(A–C)**, a near complete recovery was observed when the effects of TTX wore off and binocular vision was restored for about a week **(D–F)**. Quantification of neurofilament-positive cell density revealed no significant difference between eye-specific layers when binocular vision followed MI at 10 weeks of age (symbols connected with a solid line in **(F)**. A single animal that received about 5 days of MI at 4 weeks of age followed by binocular vision to 8 weeks (symbols connected by a dashed line in **(F)** also expressed comparable densities between eye-specific layers (3% difference) indicative of a substantial recovery. A significant loss of neurofilament labeling was produced by 3 weeks of MD initiated at 4 weeks of age **(G–I)**; however, unlike MI, there was still a significant loss of neurofilament-positive cell density when the deprived eye was opened **(J–L)** and binocular vision was provided for 2 weeks. Scale bar = 100 μm. Symbols in **(D)** represent measurements from the A (white circles) and A1 (black circles) layers, and lines connect layer-specific measurements between the eyes from the same animal. Values above the graphs represent the percentage difference between inactivated/deprived-eye layers and normal-eye layers of the dLGN. Histology images displayed in the leftmost column represent dLGN layers serving the inactivated eye (IE) or deprived eye (DE); histology images displayed to the right represent dLGN layers serving the normal eye (NE). Asterisk indicates significance less than 0.05.

As described earlier, MI for 10 days imposed at 10 weeks of age produced a reduction of neurofilament labeling within inactivated-eye layers of the dLGN (Figure 6A). Compared to layers serving the normal eye (Figure 6B), there was a 48% loss of neurofilament-positive cell density within inactivated-eye layers that was statistically significant (Figure 6C). We observed a substantial recovery of neurofilament reactivity when MI at 10 weeks of age was followed by about a week of binocular visual experience (Figures 6D,E). Although MI significantly reduced neurofilament-positive cell density, subsequent provision of binocular vision stimulated a near complete recovery so that the difference between eye-specific layers was only 8% (Figure 6F). The difference in neurofilament-positive cell density between previously inactivated-eye layers (mean = 80 cells/mm^2^; SD = 6 cells/mm^2^) and normal-eye layers (mean = 87 cells/mm2; SD = 7 cells/mm^2^) was not statistically significant (Wilcoxon test; *p* = 0.16). To assess recovery potential with MI imposed at an earlier age, we obtained data from a single animal that was reared for a separate study. This animal received one monocular injection of TTX at 4 weeks of age, then had binocular vision until it was 8 weeks old. This produced about 5 days of inactivation (Kwaw et al., 2025), and because the effect of MI is about double that of MD (Duffy et al., 2023b) we estimate this MI duration reduced neurofilament labeling by 40–50% given a comparable duration of MD elicits about a 25% loss of neurofilament (Kutcher and Duffy, 2007). Data from this animal (symbols connected by dashed lines in Figure 6F) revealed a 3% difference in neurofilament-positive density between eye-specific layers suggesting that considerable recovery can occur even when MI is imposed early in postnatal development. Closure of the lids of one eye (MD) for 3 weeks at 4 weeks of age elicited a clear reduction of neurofilament immunolabeling in the dLGN that was comparable to the loss observed after MI imposed at 10 weeks of age. Deprived-eye layers (mean = 43 cells/mm^2^; SD = 4 cells/mm^2^; Figure 6G) showed a 51% loss of neurofilament-positive cell density compared to layers serving the non-deprived eye (mean = 86 cells/mm^2^; SD = 6 cells/mm^2^; Figure 6H), which was a statistically significant difference (Wilcoxon test; *p* < 0.05; Figure 6I). Although neurofilament reactivity showed some recovery when the deprived eye was opened and binocular vision was provided for 2 weeks, deprived-eye dLGN layers (mean = 51 cells/mm^2^; SD = 4 cells/mm^2^; Figure 6J) still showed a 35% reduction of neurofilament-positive cell density when compared with non-deprived layers (mean = 79 cells/mm^2^; SD = 6 cells/mm^2^; Figure 6K), and this was a statistically significant difference (Wilcoxon test; *p* < 0.05; Figure 6L). These results indicate that, following similar losses of neurofilament, greater recovery is observed when binocular vision follows MI compared to MD.

## 4 Discussion

Results from this study reveal the profound efficacy of monocular retinal inactivation to elicit reduction of neurofilament protein within cells of the dLGN. The reduction of neurofilament within dLGN layers postsynaptic to the inactivated eye was highest when MI was imposed at 4 weeks of age, a time when visual system plasticity potential is at its highest (Olson and Freeman, 1980). With increasing age, the impact of MI on neurofilament immunolabeling diminished; however, a significant effect of MI was still measured at the oldest age examined, 22 weeks, which is well past the classical critical period for ocular dominance plasticity. Despite the large reduction of neurofilament produced by MI imposed at 10 weeks of age, a near complete recovery was measured within about a week of TTX wearing off during which binocular vision was provided. Less recovery was observed in animals that exhibited a comparable loss of neurofilament elicited by MD that were provided 2 weeks of binocular vision post MD. These results demonstrate the remarkable efficacy of retinal inactivation to elicit neural modifications within the dLGN at ages within and beyond the classical critical period, as well as a surprising capacity for recovery with provision of binocular vision after MI.

### 4.1 Potential impact of MI and MD on signal latency

The assembly of neurofilaments into a stable intracellular scaffold is believed to endow neurons with structural stability (Smith, 1973; Song et al., 2015). Neurofilament is a major protein constituent of axons where it is positively correlated with axonal caliber—thicker axons have more neurofilament (Hoffman et al., 1987; Cleveland et al., 1991). As a consequence of its link to axon caliber, neurofilament has a functional association with axon conduction velocity because large diameter axons rich with neurofilament conduct action potentials faster than thinner ones with less neurofilament (Perrot et al., 2007; Nowier et al., 2023). The significant loss of neurofilament protein within the dLGN in the context of abnormal visual experience like MI and MD raises the possibility that afferent signals from the affected eye, conducted through the dLGN, would exhibit a timing delay compared to signals transmitted from the normal fellow eye. Indeed, a timing differential is observed in the context of human amblyopia where visually-evoked potentials measured from the visual cortex exhibit longer latency for signals transmitted from the amblyopic eye (Hosseinmenni et al., 2015; Wang et al., 2025). The increase in amblyopic-eye signal latency may in part originate from a loss of neurofilament protein within dLGN neurons serving the amblyopic eye because this could reduce the axon caliber of affected neurons and thus slow conduction velocity. Interestingly, occlusion of the fellow eye as a means to remediate vision deficits in the amblyopic eye stimulates a restoration of VEP latency in humans (Barnard and Arden, 1979), and similar occlusion treatment also elicits recovery of neurofilament in the dLGN of cats subjected to an early amblyogenic MD (O’Leary et al., 2012). In the current study, restoration of neurofilament labeling with binocular vision following MI suggests that any functional effects imposed by the reduction of neurofilament are transient, which is consistent with results showing recovery of cortically measured visually-evoked potentials in cats subjected to MI that then received binocular vision (Duffy, 2025). Although visually-evoked potentials represent a sensitive means of estimating the global strength of synaptic excitation (Gavornik and Bear, 2025), future research using single-unit recordings can examine if recovered neurons are identical to controls.

### 4.2 Recovery from the effects of MI

The recovery of neurofilament labeling in the dLGN after MI is consistent with past studies that have also demonstrated anatomical recovery after retinal impulse blockade with TTX. In kittens, a significant atrophy of dLGN neuron soma size following 1 week of MI started at 7 weeks of age resolved to near normal values when binocular vision was provided after the period of inactivation (Kupperman and Kasamatsu, 1983). Within approximately 3 weeks after the week-long MI that produced a 29% atrophy of neurons postsynaptic to the inactivated eye, the size of neurons recovered so that the difference between inactivated and fellow-eye dLGN neurons was only 5% when binocular vision was provided. Likewise, cytochrome oxidase staining, an oxidative metabolic marker for neurons, exhibited a marked reduction after 4 weeks of MI in adult cats, but this resolved completely with subsequent binocular vision (Wong-Riley and Riley, 1983). Similar recovery has been observed in the visual cortex of young adult macaque monkeys that received MI for up to 15 days. While these monkeys showed no reduction in the total number of neurons following MI, there was a significant loss of GABA immunoreactivity within ocular dominance columns serving the inactivated eye. The MI-induced reduction of GABA recovered to normal levels when the effects of TTX wore off and binocular vision was restored (Hendry and Jones, 1988). These data, in aggregate with our finding that more recovery is observed after MI than MD with a comparable deficit, suggest that the alteration of visual experience produced by MD is more conducive to producing the durable deficits linked to amblyopia than the ephemeral alterations elicited by a short duration of MI. Indeed, a brief duration of MD results in greater synaptic depression of deprived-eye responses compared to brief MI (Rittenhouse et al., 1999) indicating that the residual retinal activity with MD facilitates weakening of deprived-eye connections, and that the absence of activity with MI is protective against these changes (Blais et al., 1999). Future studies will need to determine if longer durations of MI produce indelible modifications within the visual system that associate with amblyopia, and especially whether such durations of MI initiated during the early stages of visual system development produce intractable changes within the primary visual pathway.

Data from the current study were clear in revealing greater recovery of neurofilament labeling when a period of binocular vision followed MI compared to MD, even though the magnitude of neurofilament loss was comparable between the two forms of visual deprivation. In considering MI as a potential treatment for amblyopia (Duffy et al., 2018; Fong et al., 2021) this is potentially meaningful because it suggests that temporary inactivation of the fellow (non-amblyopic eye) is less likely to inadvertently produce iatrogenic amblyopia. Although often transient, reduced vision in the fellow eye due to full-time patching is estimated to occur in 19% of patients and is most frequent at early ages (Longmuir et al., 2013). Importantly, the period of MD used in the current study was initiated earlier in development and lasted longer than the period of MI. Because MI elicits effects in the dLGN that are much larger than those produced by a similar duration of MD applied at the same age (Duffy et al., 2023b), the current study created comparable effects between the two forms of deprivation by imposing shorter durations of MI. To fully investigate whether the effect of MI is inherently more reversible than MD, it will be important to systematically compare recovery from the two forms of visual deprivation by investigating age-matched animal pairs across different stages of development and following different durations of deprivation.

### 4.3 Homeostatic and Hebbian plasticity

The visual disturbances produced by MD and MI are different. Visual deprivation by lid closure attenuates the transmission of light entering the deprived eye but some visual perception is preserved (Crawford and Marc, 1976). Cats subjected to binocular lid closure are able to learn and perform light/dark behavioral discriminations with the lids still closed (Loop and Sherman, 1977), and about a third of neurons in the primary visual cortex respond to visual stimuli presented through the closed eyelids (Spear et al., 1978). On the other hand, retinal inactivation with TTX produces a different disturbance in which retinal output activity is abolished thereby eliminating visually-evoked cortical responses and also sensory-driven visual perception (Fong et al., 2016; Fong et al., 2021). The impact on cortical plasticity is also different between the two forms of visual deprivation with MD eliciting greater synaptic depression compared to MI (Rittenhouse et al., 1999). In the current study, the superior recovery of neurofilament labeling after MI may partly derive from a difference in the constellation of plasticity mechanisms engaged by the two forms of deprivation. With MD, residual activity from the closed eye is thought to produce an interocular imbalance suited to drive weakening of deprived-eye synapses that can yield a durable shift in cortical ocular dominance via mechanisms of Hebbian plasticity (Blais et al., 1999). A deeper impact on cortical neural connections may diminish the potential for recovery following MD. The elimination of retinal output activity with MI may elicit homeostatic plasticity mechanisms, such as an increase in glutamate receptors, that are engaged to compensate for the profound loss of afferent activity and consequently protect against the loss of synapses during the period of inactivation (Stellwagen and Malenka, 2006). The profound post-synaptic reduction of dLGN neuron soma size (Kupperman and Kasamatsu, 1983) and the loss of neurofilament protein elicited by MI may in part arise from a transient state of dystrophy induced by a loss of retinal-derived trophic support (Mandolesi et al., 2005). Once the inactivation has abated, restored retinal activity could stimulate the recovery of these features.

### 4.4 Implications for amblyopia treatment

Recovery from the effects of early MD with inactivation of the dominant fellow eye has emerged as a potential approach to remediate deprivation amblyopia (Duffy et al., 2018; Fong et al., 2021; Kwaw et al., 2024), and to our knowledge it does not produce detriment to the inactivated eye (Foeller and Tychsen, 2018; DiCostanzo et al., 2020; Hogan et al., 2023; Kwaw et al., 2025). In this study, we found that MI resulted in a profound reduction of neurofilament within layers of the dLGN serving the inactivated eye. Although the magnitude of the loss declined as MI was imposed at later ages, we continued to observe a significant effect of MI even well past the classical critical period for ocular dominance plasticity (Olson and Freeman, 1980). In the context of amblyopia treatment in which the fellow dominant eye is administered TTX, the decrease in neurofilament during the period of inactivation may act to alleviate imbalances between the eyes, including signal conduction velocity, that facilitate recovery of neurons serving the amblyopic eye. This raises the intriguing possibility that pharmacologic retinal inactivation may exhibit efficacy to treat human amblyopia at ages when occlusion therapy fails to promote benefit. Further, the measured recovery of neurofilament following MI implies that its plasticity-promoting effects do not precipitate significant long-term damage to neurons or neural connections within the primary visual pathway.

## Data Availability

The raw data supporting the conclusions of this article will be made available by the authors, without undue reservation.
